# Striking Denervation of Neuromuscular Junctions without Lumbar Motoneuron Loss in Geriatric Mouse Muscle

**DOI:** 10.1371/journal.pone.0028090

**Published:** 2011-12-02

**Authors:** Ruth Jinfen Chai, Jana Vukovic, Sarah Dunlop, Miranda D. Grounds, Thea Shavlakadze

**Affiliations:** 1 School of Anatomy and Human Biology, The University of Western Australia, Perth, Western Australia, Australia; 2 Queensland Brain Institute, The University of Queensland, St. Lucia, Queensland, Australia; 3 School of Animal Biology, The University of Western Australia, Perth, Western Australia, Australia; University of Houston, United States of America

## Abstract

Reasons for the progressive age-related loss of skeletal muscle mass and function, namely sarcopenia, are complex. Few studies describe sarcopenia in mice, although this species is the mammalian model of choice for genetic intervention and development of pharmaceutical interventions for muscle degeneration. One factor, important to sarcopenia-associated neuromuscular change, is myofibre denervation. Here we describe the morphology of the neuromuscular compartment in young (3 month) compared to geriatric (29 month) old female C57Bl/6J mice. There was no significant difference in the size or number of motoneuron cell bodies at the lumbar level (L1–L5) of the spinal cord at 3 and 29 months. However, in geriatric mice, there was a striking increase (by ∼2.5 fold) in the percentage of fully denervated neuromuscular junctions (NMJs) and associated deterioration of Schwann cells in fast extensor digitorum longus (EDL), but not in slow soleus muscles. There were also distinct changes in myofibre composition of lower limb muscles (tibialis anterior (TA) and soleus) with a shift at 29 months to a faster phenotype in fast TA muscle and to a slower phenotype in slow soleus muscle. Overall, we demonstrate complex changes at the NMJ and muscle levels in geriatric mice that occur despite the maintenance of motoneuron cell bodies in the spinal cord. The challenge is to identify which components of the neuromuscular system are primarily responsible for the marked changes within the NMJ and muscle, in order to selectively target future interventions to reduce sarcopenia.

## Introduction

The precise reasons for the age-related loss of muscle mass and function, known as sarcopenia, are not well understood [1], [2]. The incidence of sarcopenia determined by dual-emission X-ray absorptiometry to measure skeletal muscle mass is reported as 14% in humans aged 65–69 years and >50% in those 80 years or older [3]. For specific muscles, the extent of mass loss may reach ∼20–30% for the limb muscles and up to ∼40% for the trunk muscles between 68 and 100 years [4]. Similarly, in female mice, a 30% quadriceps muscle mass loss occurs between 15 to 29 months [5] (corresponding roughly to 60–80+ years in humans) (http://research.jax.org/faculty/harrison/ger1vLifespan1.html).

Age-related changes in skeletal muscles are complex with key features being myofibre atrophy and death, disruption of the contractile apparatus, changes in extracellular matrix composition and deterioration of neuromuscular junctions (NMJs) leading to functional denervation of the ageing muscle [1], [4], [6]. These changes in ageing muscle involve interactions between many systemic and local factors [7]. To date, most studies on sarcopenia have focused on alterations in muscle protein turnover, anabolic resistance to feeding [8], [9], [10] and stem cells [5], [11], with vital interactions between nerves and muscles being largely overlooked.

Neuromuscular changes contributing to myofibre denervation occur within the central and peripheral nervous systems as well as within skeletal muscle tissue. Changes include diminished function or loss of neurons in the brain and spinal cord, demyelination of nerves and progressive degeneration of NMJs [1], [4], [12], [13]. The vertebrate NMJ is composed of the presynaptic nerve terminal, the postsynaptic specialised membrane of the myofibre, plus Schwann cells (SC) that envelope nerve axons and terminal branches that intermittently extend fingers into the synaptic cleft [14]. A number of changes have been documented during ageing including (i) a loss of motoneuron numbers in the central nervous system (CNS) [13], [15]; (ii) demyelination of axons [16]; (iii) withdrawal of nerve-terminals from the NMJs [17]; and (iv) some axonal sprouting and re-innervation of denervated myofibres by surviving motoneurons [4]. However, it is not known whether the initial myofibre denervation is related to deleterious changes in muscle cells themselves or neurons or both components. However, it seems that the maintenance of NMJs depends on the healthy state of motoneurons, myofibres and other cells and the exchange of trophic signals by these cells [1], [18], [19]. Morphological changes at the NMJs are well described in old humans [20], [21] and rats [22], [23], but only very recently in old mice [17], [24]. The paucity of mouse data is largely due to the general absence of commercially available geriatric mice. However, we were recently able to obtain geriatric mice and here establish baseline data for sarcopenia.

We selected young 3 month and geriatric 29 month old C57Bl/6J mice since these ages approximate to 20 and 80 years respectively in humans [25]. Here we report on (i) motoneuron cell bodies in the lumbar segment of the spinal cord; (ii) neuromuscular junctions with pre- and postsynaptic endplates and Schwann cells and (iii) myofibres in the lower limb muscles: tibialis anterior (TA), extensor digitorum longus (EDL) and soleus.

## Results

### No loss of α-motoneurons

**Figure 1:**
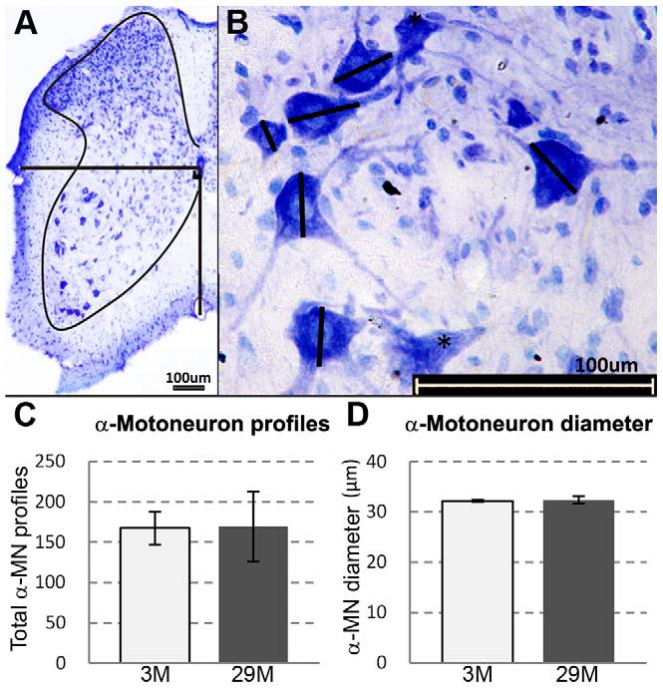
Lumbar spinal cord α-motoneurons. α-motoneurons stained with toluidine blue in the ventro-lateral quarter of the spinal cord between the bold lines were counted (A). The maximum diameter of α-motoneurons was obtained by measuring the longest axis through the nucleolus (B). Motoneurons with no visible nucleolus (*) or with diameters <25 µm were not included. Total number of α-motoneuron profiles (C) and average diameter (D) of α-motoneurons were analyzed in 20 sections of a 1 in 20 series of the lumbar region (L1–L5) in spinal cords for each mouse. There was no significant change in the average number (C) and diameter (D) of α-motoneurons between mice aged 3 and 29 months. N = 4 mice per age group. Values are mean ± s.e.m.

Analysis of the α-motoneurons (neurons with a diameter ≥25 µm) in the lumbar region (L1–L5) of the spinal cord sampled at 3 and 29 months showed no significant difference in the average diameter or number of α-motoneuron profiles (Fig. 1).

### Altered NMJs in aged EDL and soleus

Confocal images of presynaptic nerve terminals (detected with synaptophysin) and postsynaptic endplates (detected with α-bungarotoxin which is specific for acetylcholine receptors) enabled visualisation of the morphology and innervation of NMJs in EDL and soleus muscles.

**Figure 2:**
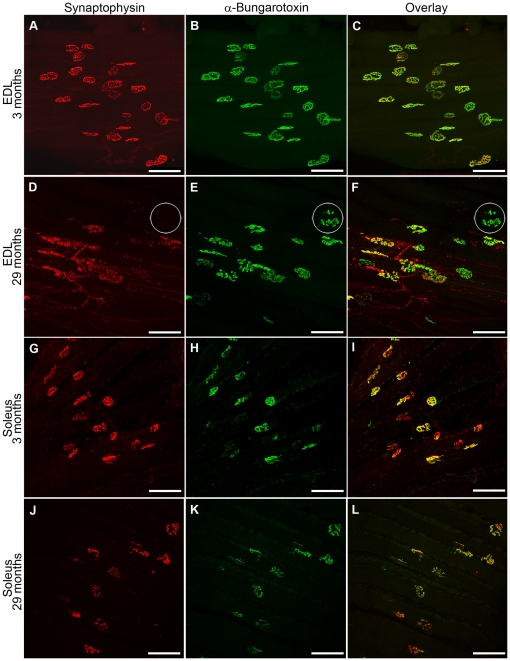
Whole mount immunohistochemical preparations of EDL (A–F) and soleus (G–L) muscles from 3 and 29 month old mice. Muscles were stained with synaptophysin (A,D,G,J; red) to detect pre-synaptic neuronal compartments and with α-bungarotoxin (B,E,H,K; green) to detect acetylcholine receptors at the muscle endplates. Overlays are shown in (C,F,I,L; yellow). Muscle endplates that are positive for only α-bungarotoxin (green) are not innervated. One such endplate is indicated (white circle) in the 29 month old EDL (D,E,F). NMJs in the 3 month old EDL appear compact and well defined (A–C), while many NMJs have a diffused, irregular and fragmented appearance in the 29 month old EDL (D–F). In contrast, the NMJs in soleus of geriatric mice (J–L) did not show morphological changes when compared to 3 month old NMJs (G–I). Scale bars are 75 µm.

**Figure 3:**
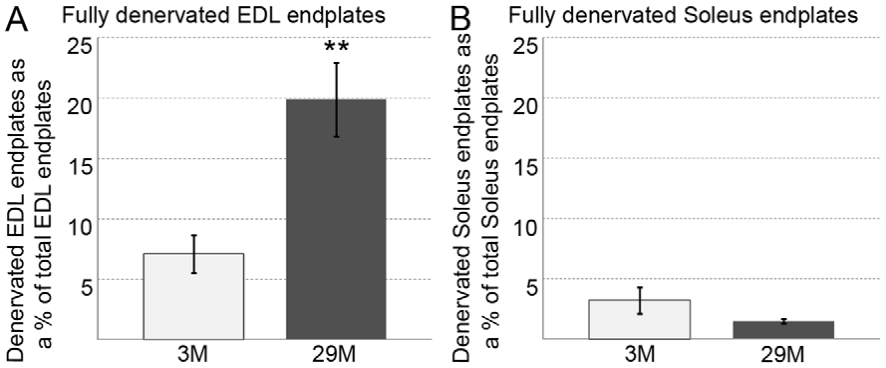
Percent of fully denervated NMJ in EDL (A) and soleus (B) muscles from 3 and 29 month old mice. There was a significantly increased number of fully denervated endplates in geriatric EDL (A) but not soleus (B) muscles. N = 4 mice per age group. **P<0.005. Values are mean ± s.e.m.

At 3 months in the EDL, both presynaptic nerve terminals and postsynaptic motor endplates were well organised, distinct and compact (Fig. 2A–C). However, by 29 months, significant remodelling had occurred, within both compartments of the NMJs (Fig. 2D–F). At 29 months, presynaptic nerve terminals were disorganised, with extensive sprouting, and some nerve terminals had spherical, enlarged ends (Fig. 2D). Postsynaptic endplates appeared diffuse, irregular and with granular fragmentation (Fig. 2E). In addition, geriatric NMJs appeared larger in diameter and more spread out compared to those in young mice. NMJs located in different areas within the same muscle showed various degrees of deterioration, with ‘healthy’ looking NMJs located in close proximity to disorganised and partially or fully denervated ones. As an example, we show a muscle area with severely disorganised NMJs in the EDL of a 29 month old mouse (Fig. 2D–F). In addition, within the same area, we identified some NMJs that were clearly denervated since they stained only with BTX and lacked presynaptic staining with synaptophysin (circled in Fig. 2D–F). Quantitation revealed a ∼2.5 fold increase in completely denervated NMJs in 29 month old EDL compared to 3 months (Fig. 3A).

The NMJs of soleus in geriatric mice (Figs. 2J–L) did not have the same striking morphological changes observed in the geriatric EDLs. Quantitation revealed no significant change in the number of totally denervated NMJs in 29 month old soleus compared to 3 months (Fig. 3B).

### Altered Schwann cells in old EDL muscles

**Figure 4:**
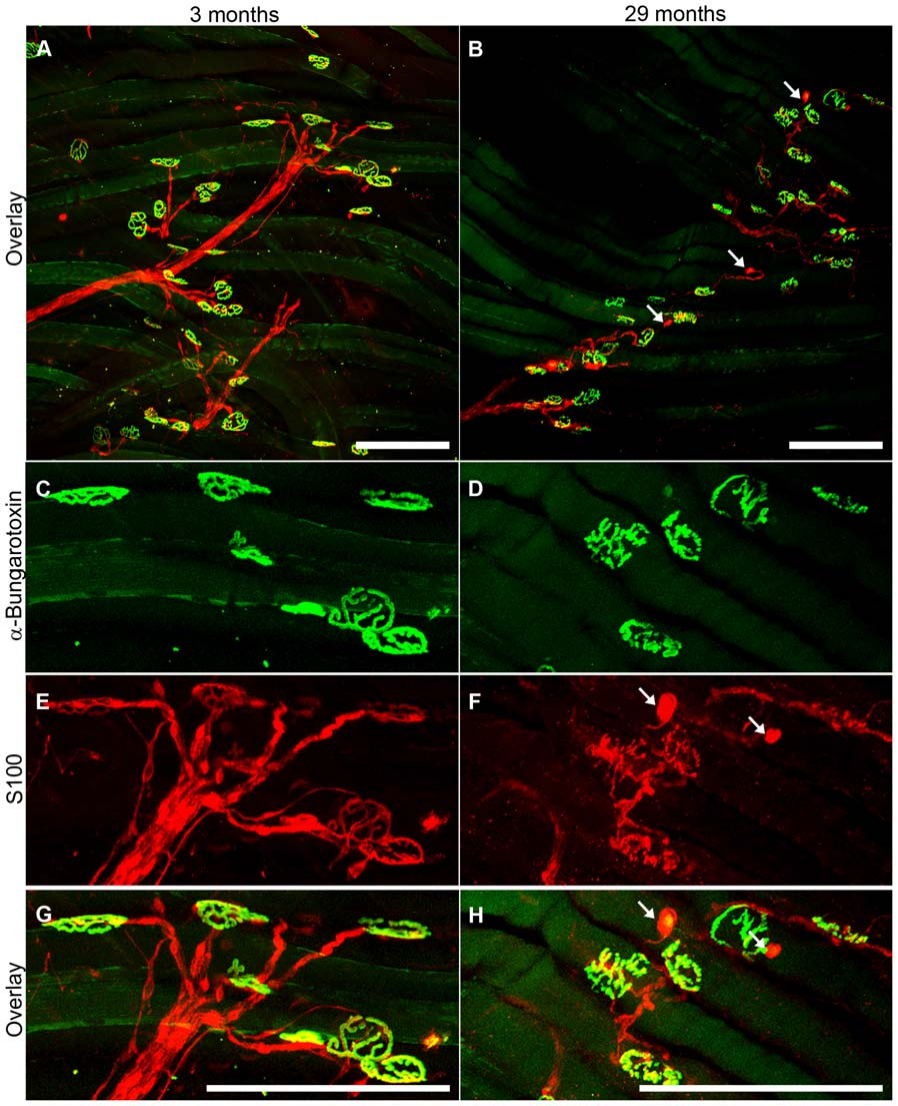
Age-related changes in the Schwann cells (SCs) at the NMJs. Low (A,B) and high (C,D,E,F,G,H) power views. Confocal images of NMJs and SCs in the EDLs of 3 (A,C,E,G) and 29 (B,D,F,H) month old mice. High power views show that SCs of young mice are structured and completely overlay with the muscle endplates (C,E,G). SCs of old mice are disorganised, partially cover muscle endplates and have swollen endings (white arrows) (D,F,H). Scale bars are 150 µm.

Schwann cells (SCs) surround and insulate peripheral axons and have close interactions with the nerves and endplates at the NMJs. SCs in EDL muscles were visualised with S100 antibody in 3 and 29 month old mice. At 3 months, SCs were well structured, ‘plump’ and were in contact with the entire endplate (Fig. 4A,C,E,G). At 29 months, SCs were disorganised, thinner and only partially covered the endplates (Fig. 4B,D,F,H).

### Phenotypic characterisation of young and old mice and their muscle weights

Tibial bone length increased with age, being significantly longer at 29 months compared with 3 months (Table 1), indicating some linear growth. Total body weight, and body weight standardised to tibial length, increased respectively by 45% and by 25% between 3 and 29 months (Table 1). Total quadriceps weight was similar in young and old mice. By contrast, quadriceps weight standardised to tibial length decreased by 23% in old mice (Table 1). Total TA weight was similar in young and old mice; however, TA weight standardised to tibial length decreased by 22% between 3 and 29 months (Table 1). Total and standardised weights of EDL and soleus muscles were similar in young and old mice (Table 1). There was no significant difference in the amount of abdominal fat between 3 and 29 month old mice, which may be due to large variation within the aged group (Table 1).

**Table 1:** Phenotypic characterisation of 3 and 29 month old mice: tibial bone length, body weight, limb muscle weights and abdominal fat pad weight.

Age	3 months	29 months
Number of mice per group	6–20	7–13
Tibial length (TL)(cm)	1.61±0.01	1.83±0.02**
Body weight (g)	19.43±0.24	28.27±1.54**
Body weight/TL (g/cm)	12.22±0.18	15.33±0.96**
Quad weight (mg)	124.23±3.95	114.08±4.04
Quad weight/TL (mg/cm)	79.29±3.22	61.13±2.16**
TA weight (mg)	34.83±1.38	31.50±0.89
TA weight/TL (mg/cm)	21.88±1.66	17.03±0.49**
EDL weight (mg)	7.25±0.50	7.88±0.39
EDL weight/TL (mg/cm)	4.55±0.48	4.28±0.23
Soleus weight (mg)	5.42±0.64	6.36±0.37
Soleus weight/TL (mg/cm)	3.39±0.40	3.41±0.20
Abdominal Fat Pad (g)	0.17±0.02	0.54±0.14

For body and muscle weights absolute values as well as values standardised to tibial bone length are shown.

**P<0.05. All values are mean ± s.e.m.

### Myofibre number and size in EDL and soleus muscles

**Figure 5:**
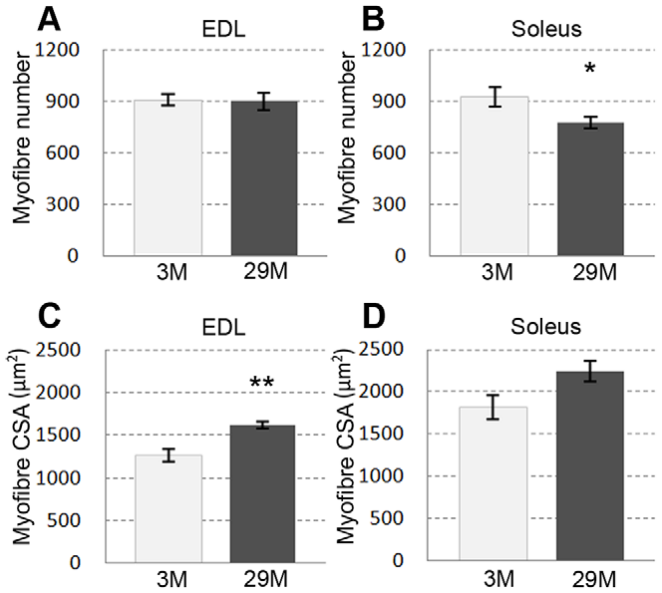
Total myofibre number (A,B) and average myofibre cross-sectional area (CSA) (C,D) in EDL and soleus muscles. At 29 months, there was no significant loss of myofibres in the EDL (A), but a significant loss in the soleus (B). The average myofibre CSA was larger in 29 month old compared to 3 month old EDLs (C); whereas the average myofibre CSA was similar in soleus muscles at 3 and 29 months (D). N = 4 mice per age group. *P<0.05, **P<0.005. Values are mean ± s.e.m.

There were approximately 900 myofibres in the cross-section through the mid-belly of the EDL and soleus muscles at 3 months (Fig. 5A,B). In the EDL, there was no change in the number of myofibres between young and old mice (Fig. 5A). However, the average myofibre cross sectional area was larger by 27.6% at 29 months compared to 3 months (Fig. 5C). In the soleus, myofibre number was reduced by 16% at 29 months compared to 3 months (Fig. 5B) whereas myofibre cross sectional area was not significantly different (Fig. 5D).

### Myofibre types and cross-sectional area in TA, EDL and soleus muscle

**Figure 6:**
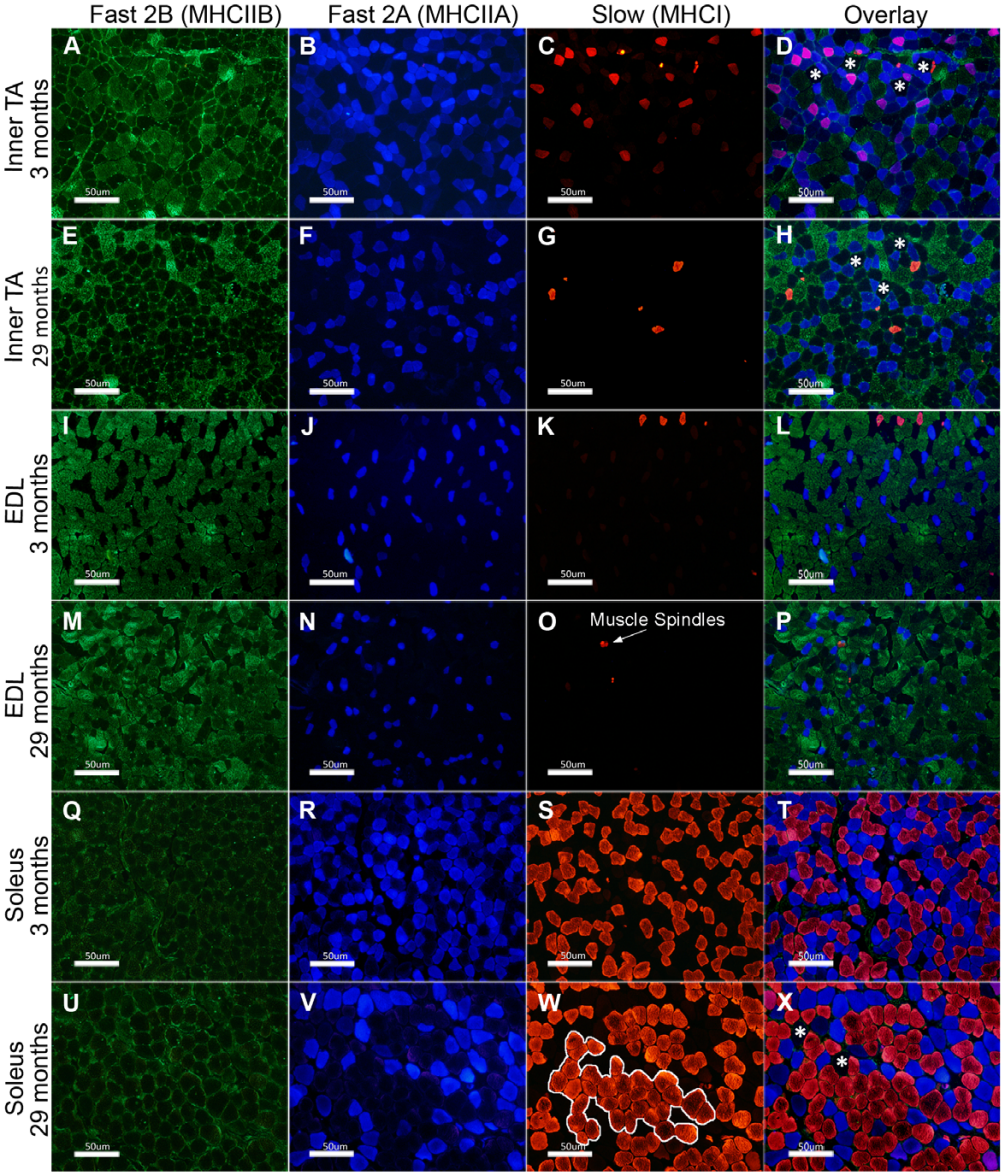
Fast 2B, Fast 2A and slow myofibres in the inner TA, EDL and soleus muscles. Antibodies for MHCIIB, MHCIIA and MHCI were used to detect three different types of myosin respectively: fast 2B (A,E,I,M,Q,U), fast 2A (B,F,J,N,R,V) and slow (C,G,K,O,S,W). The overlay of these is shown in D,H,L,P,T and X. Myofibres not detected with either of these antibodies were presumed to be fast 2× (MHCIIX) (a few are indicated by asterisks * in D, H, X). Along with the slow type myofibres, antibody for MHCI also stains muscle spindles (arrow in O). Grouping of slow type 1 myofibres was seen in 29 month soleus muscles (outlined in W). Scale bars are 50 µm.

**Figure 7:**
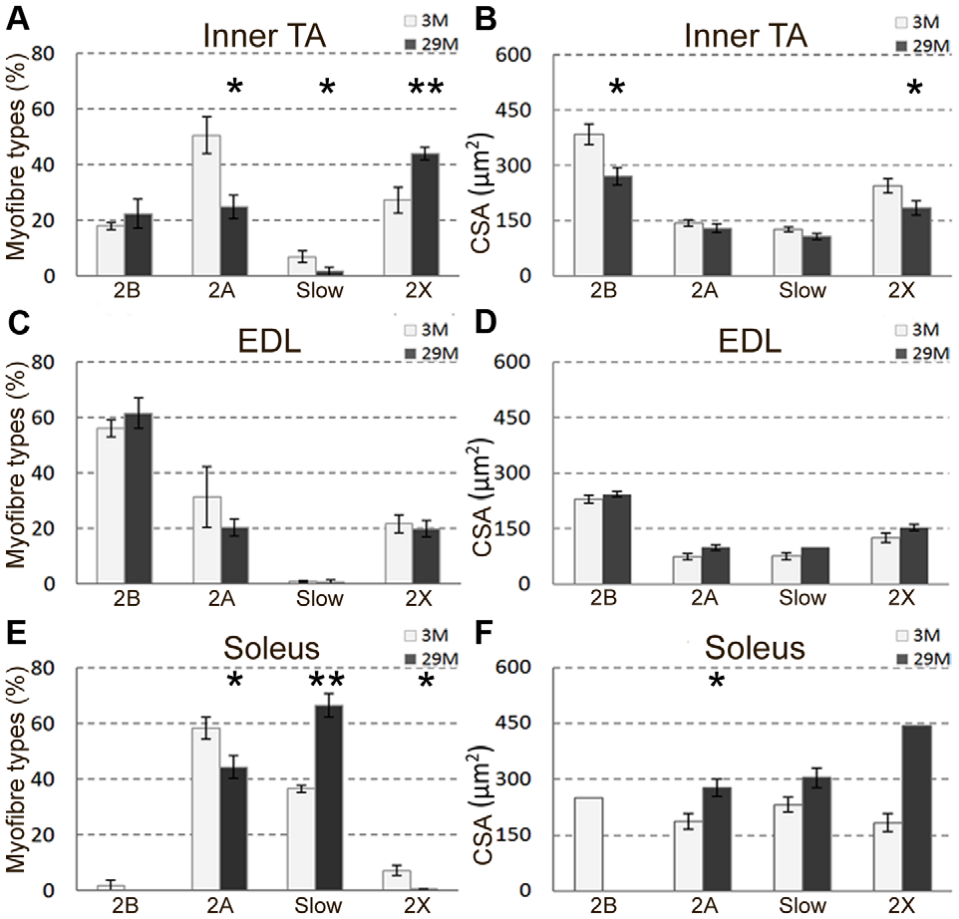
Percentage (A,C,E) and average cross-sectional area (B,D,F) of different myofibre types in the TA, EDL and soleus muscles of 3 and 29 month old mice. There are no error bars on some of the graphs (D,F) as the myofibre types were present in less than 3 animals (i.e. variation in myofibre type distribution occurred in different animals within the same age group). N = 4 animals per age group. *P<0.05, **P<0.005. Values are mean ± s.e.m.

The myofibre types in the inner portion (close to the bone) of the TA, and the entire transverse section of EDL and soleus muscles were analysed using antibodies specific to the slow (MHCI), fast 2A (MHCIIA) and fast 2B (MHCIIB) myosins (Fig. 6). Unlabelled myofibres were presumed to contain fast 2× (MCHIIX) myosin [26]. Quantification of number and cross sectional area of these different myofibre types in the TA, EDL and soleus muscles are shown in Fig. 7. Note that the total percentages of myofibre types do not always add up to 100% as some myofibres co-express more than one MHC isoform.

#### TA (Inner portion)

At 3 months, the inner portion of TA muscles was composed of fast 2B (18%), 2× (27%) and 2A (50%), and slow (7%) myofibres (Figs. 6A–D, 7A). At 29 months, there was a 50% loss of fast 2A and 76% loss of slow myofibres, and a 38% increase of fast 2× myofibres (Figs. 6E–H, 7A). Cross sectional area of fast 2B and fast 2× myofibres was smaller by 30% and 25% respectively at 29 months compared to 3 months, with no change in the CSAs of the fast 2A or slow myofibres (Fig. 7B).

#### EDL

At 3 months, EDL muscles were composed of fast 2B (56%), fast 2× (22%), fast 2A (31%) and occasional (less than 1%) slow myofibres were also seen (Figs. 6I–L, 7C). There was no significant change in the number and cross sectional area of different myofibre types (Fig. 7C,D).

#### Soleus

At 3 months, the majority of myofibres in soleus were fast 2A (58%) followed in number by slow (36%) and fast 2× (7%) (Figs. 6Q–T, 7E). Slow myofibre type grouping was seen in 29 month old soleus muscles (Fig. 6W), which is indicative of reoccurring denervation and reinnervation in the ageing muscle [4]. There was a 24% loss of fast 2A and a 45% increase in slow myofibres at 29 compared to 3 months (Fig. 7E). The cross sectional area of fast 2A myofibres was larger by 32%, and there was a trend towards larger slow myofibres at 29 months compared to 3 months (Figs. 6U–X, 7F). The soleus muscle generally did not have fast 2B myofibres (Fig. 6Q,U), but one of the 3 month old soleus (out of 4 analysed) had a few fast 2B, while one 29 month old soleus (out of 4 analysed) had a few fast 2× myofibres.

## Discussion

The major findings of this study are that, whereas α-motoneurons in the spinal cord of mice do not change either their size or number with ageing, profound changes are seen at the neuromuscular junction. Disruptions are seen both within the pre- and postsyanaptic compartments as well as in the terminal Schwann cells in the EDL. By contrast, pre- and postsynaptic compartments appear relatively intact in the soleus muscle. These changes are mirrored by a striking increase in the number of completely denervated NMJ in EDL, but not soleus. Despite the fact that soleus muscles did not show an age-related response of the NMJs, the myofibre composition of the soleus showed age-related plasticity, as did the TA.

### No loss of α-motoneurons in geriatric mice

In our study, the size and numbers of α-motoneurons were determined in the lumbar region (L1–L5) of young and geriatric mice. The lumbar area was selected since, in humans and rats, sarcopenia is more dramatic in lower limb muscles compared to the upper body muscles [23], [27], [28]. Also, studies in rats show that age-related axon degeneration is more prevalent in ventral roots and peripheral nerves initiating from the lumbar compared to the cervical level of the spinal cord [29], [30]. Our inclusion of L1–L5 reflects the fact that, in mice, the sciatic nerve originates from motoneurons located in the L3–L5 region as determined by retrograde labelling [31].

Some studies in mice suggest that there is a loss of motoneurons in ageing [17], [32], while others do not [33]. In our study, the diameter and average number of α-motoneuron profiles in the lumbar segment of the spinal cord did not differ between 3 and 29 months, indicating that α-motoneuron cell bodies do not die as a result of ageing. Conflicting data reported by others for motoneuron numbers in old mice may be due to the methods used to quantify motorneurons, with some studies counting myelinated axons only without counting motoneuron cell bodies [17], [33], [34]. Indeed, it has been shown that cortical motorneurons with disconnected axons can persist for as long as a year after axotomy [35]. Another limitation to using myelinated motor-axon counts to quantify motoneuron numbers is that aged motor-axons frequently show extensive atrophy and demyelination, with such degenerative changes being more severe in the distal compared to the proximal part [36] and these demyelinated motor-axons will be omitted from the total axonal count [37]. Similar to the mouse, data regarding motoneuron loss in aged rats are also conflicting. However, strain differences in rats may account for this disparity. Although one study reported no change in the number of motor-axons innervating the soleus in 24 month old rats (strain not specified) [34], another revealed a 45% decrease in motoneuron cell bodies in lumbar (L4/L5) region of 22 month male Fischer 344 rats [38]. In humans, based on motoneuron cell body counts in the lumbosacral segment of the spinal cord, ∼29% of motoneurons are lost in the seventh decade [15]. This marked loss of motoneuron cell bodies in humans may reflect the very long absolute time, ∼10–20 years, that the axon is disconnected from the target myofibre, compared with only months in rodents. The extent of age-related loss of motorneuron cell bodies remains unclear for different species. Interestingly, in several species including humans, stereological assessment of the neocortex and hippocampus led to the somewhat surprising conclusion of minimal age-related loss of neuron cell body number, indicating that central neuronal degeneration is not significantly involved in normal ageing even though the function of the ageing CNS is compromised [39].

Although our data show no change in the size or number of α-motoneuron profiles in ageing mice, the function of surviving α-motoneurons may be deficient. A report on aged monkeys with cognitive impairment but no neuron loss, suggested that connections to and from the prefrontal cortex were intact but functionally compromised [40]. Changes reported in the morphology of dendritic arbors, spines, and synapses of rodents may impact on the function of hippocampal circuits but would not be reflected as neuron loss *per se* [39].

### Altered NMJs in old EDL and soleus muscles

The ultimate indication of myofibre denervation in ageing muscle is whether the nerve is connected to the myofibre. We therefore counted the number of fully denervated NMJs, but did not include partially denervated or morphologically altered NMJs. We showed a ∼2.5 fold increase (up to ∼20%) of fully denervated endplates in EDL muscles between 3 and 29 month, but no change in the soleus. Only two other studies have examined myofibre denervation in ageing mice, although they used different techniques. In flexor digitorum brevis muscle of 21–24 month old FVB mice, electrophysiological analysis of sodium current combined with immunostaining for sodium channels revealed that 13% of myofibres were fully denervated [41]. In another study using Thy1-XFP transgenic mice that express green fluorescent protein in motoneurons (C57BL/6J background), 15% of myofibres were denervated in TA muscles at 24 months [17]. Similarly, in EDL muscles of 32 month old rats (WI/HicksCar), ∼17% of myofibres were denervated [22].

Although we did not include partially denervated myofibres in our analysis, others have reported that ∼35% of endplates are partially denervated in the limb muscles of 21–24 month old mice [17], [41]. Taken together, these combined studies show that ∼50% of myofibres in some, but not all, limb muscles of ageing mice can be partially or fully denervated by 24 months.

We found that although such denervation was pronounced in the EDL it was not apparent in the soleus. Such marked variation between muscles have also been described for aged rats [42]. Although we did not observe the increase in fully denervated NMJs in old soleus muscles, the increased proportion of slow type myofibres and myofibre type grouping in these muscles indicate reoccurring myofibre denervation and re-innervation [4]. Since soleus is a postural muscle in mice and is used more than EDL, the denervated NMJs may be efficiently re-innervated. This activity-related influence may be the reason for the sparing of NMJs in old soleus muscles as shown in 21 month old rats [42].

### Altered Schwann cell (SC) morphology with age

Although SCs are known to be important for the maintenance of innervation and regulation of re-innervation at NMJ [43], the role of SC degeneration in ageing muscles is not understood. We do not know whether the disorganisation and loss of SCs we observed is a cause or an outcome of myofibre denervation in geriatric muscle. However, such changes in SCs will have an adverse effect on the maintenance of the remaining NMJ and possible re-innervation. Most studies have examined alterations in SCs after nerve crush or transection [44], whereas very few have examined SCs in ageing muscles [45], [46]. An electron microscopic study of gastrocnemius muscles in mice showed no SC degeneration in young adults (6 months) but degeneration of SCs and their processes in 35% of the NMJs at 27 months [45]. Similarly, in biopsy samples taken from the external intercostal muscles of 17 human patients aged >70 years [46], electron microscopy showed abnormalities in SCs with their processes intruding into the primary synaptic cleft (the gap between the terminal axon membrane and the myofibre plasmalemma), while this was absent in children (4–8 years).

Schwann cells contribute to the long-term stability of NMJs by releasing neurotrophins such as nerve growth factor (NGF), brain-derived neurotrophic factor (BDNF) and ciliary neurotrophic factor (CNTF) [47]. In cases of partial muscle denervation, relevant to ageing, SCs detect reduced synaptic activity and guide nerve sprouts from innervated NMJs to denervated endplates [14].

### Possible reasons for age-dependent changes in NMJ morphology

The molecular changes that lead to age-related myofibre denervation and problems with re-innervation of NMJs are not known (discussed in [1]). Maintenance of NMJs depends on trophic factors and signals from several cell types and these may be altered by reduced electrical activity which destabilises the NMJ and results in subsequent detachment of the axon terminal from the myofibre surface. While microarray analyses reveal changed gene expression at the motor unit level, this generally seems to be the result, rather than the cause, of denervation [1], [2], [48]: a discussion of such molecular changes is beyond the scope of this paper.

Other contributing factors to myofibre denervation include oxidative stress that greatly accelerates age-related loss of NMJs [24], [43] and is strongly associated with muscle wasting in many situations [49]. Exercise and caloric restriction have been shown to reduce age-related muscle denervation in mice [17] and reduced oxidative stress may be a common mechanism underpinning the beneficial effect of these two interventions [50]. The benefits of exercise extend beyond reduced oxidative stress and include increased neurotrophic factors and growth hormones as well as increased frequency of molecular signal transmission from nerve to muscle, all of which may ameliorate the effects of ageing [1], [30].

### The extent of sarcopenia differs between various limb muscles

We standardised muscle weights to tibial bone length in order to determine age-related changes,, because linear growth (as indicated by increased tibial length) occurs between 3 to 29 months [5]. We previously described changes in quadriceps muscle mass and tibia length in ageing C57Bl/6J females using four time points, 3, 15, 24 and 27–29 months. In these mice, standardised quadriceps muscle weight was greater at 15 compared to 3 months, but decreased thereafter and by 27–29 months sarcopenia was pronounced [5]. The current study compared only 3 and 29 month old mice and showed that both quadriceps and TA muscle weights were significantly reduced at 29 months, but that EDL and soleus weights were similar at these ages. Therefore, although we might have detected reduced muscle mass in EDL and soleus if intermediate ages (e.g. 15 months) had been included, another explanation is that sarcopenia affects some muscles more than others as shown previously in 129/Re mice [51].

### Age-related changes in myofibre number and CSA

We showed a ∼16% decrease in myofibre numbers in soleus muscles between 3 and 29 months but no change in EDL. Similarly, a decrease in myofibre numbers has been reported in some muscles of aged rats and humans [34], [52]. Although we found a trend for increased average myofibre CSA in soleus, the trend was not significant, whereas myofibre CSA was significantly larger in EDL muscles at 29 months. Whereas some studies show decreased myofibre number and/or changes in the myofibre size in old mouse EDL and soleus muscles, others do not and it seems that these discrepancies are due to the use of different strains or gender [51], [53]. For instance, in contrast to our study, no myofibre loss or changes in myofibre size were seen in EDL or soleus muscles of male CBF mice aged 29 months [53], and a very recent study in C57Bl mice aged 6 and 24 months showed age-related myofibre loss in both EDL and soleus muscles with this being greater for females (∼20%) compared with males (∼10%) although myofibre numbers did not change in sternomastoid or cleidomastoid muscles [54]. Similarly, although a 16–21% loss of myofibres was reported in both EDL and soleus muscles of 24 month old female 129/Re mice, with increased size of remaining myofibres, the loss or changes in myofibre size were not seen in the same muscles of 24 month old males [51]. These data accord with the impact of gender on many aspects of skeletal muscle biology including age-related changes in skeletal muscles, with sarcopenia being less pronounced in males [30].

In ageing, heterogeneous myofibre denervation occurs [32] and this is very different to homogeneous myofibre denervation that occurs after experimental nerve transaction [55]. In aged muscles, the presence of many innervated myofibres may provide mechanical (e.g. provide passive stretch) and molecular stimulation/support to sustain adjacent denervated (‘passenger’ or ‘free-loader’) myofibres embedded in their midst.

As discussed for motoneuron cell bodies in the spinal cord, the greater reduction in myofibre numbers observed in humans compared to mice may be due to the absolute length of time that the muscles are denervated. In vastus lateralis muscles of men aged 70–73 years, myofibre number decreased by ∼25% with a further 50% decrease by 80 years [52]. However, in humans, NMJ degeneration is apparent by 50–60 years [46] and hence myofibres may be denervated for 20–30+ years.

### Variation in myofibre type composition between young and old muscles

Our observation of variation in myofibre type composition in TA, EDL and soleus at 3 months accords with the literature. Thus, in young mice, the outer part of the TA is made up almost entirely of fast 2B myofibres whereas in the inner region of the TA, fast 2B myofibres make up only 20% and the dominant myofibre type is fast 2A, with some slow myofibres [56]. The EDL is composed of 99% fast myofibres (with 60% fast 2B) with few to no slow myofibres [57]. The soleus comprises 60% fast 2A and 40% slow myofibres (Figs. 6R–T, 7E) [58] and in our 3 month mice, we saw a few fast 2B myofibres in one, but not in 3 other young soleus muscles.

In addition, we showed that the proportions of myofibre types changed with age. At 29 months, myofibre types of the inner region of the TA had shifted towards a faster phenotype, with an increase in the content of fast 2× myofibres, a decrease in fast 2A myofibres and a near complete loss of slow myofibres. In terms of size change for different myofibre types in the TA, fast 2B and fast 2× myofibres showed significant atrophy. Since the TA is made up of ∼90% fast 2B myofibres in the superficial region and ∼20% in the inner region, this significant reduction in size of fast type myofibres may account for the overall loss of TA muscle mass at 29 months. Despite our observations of a 2.5 fold increase in the number of fully denervated NMJs in EDL muscles of 29 month old mice, we did not see changes in myofibre type composition.

In contrast to TA, in the soleus, a shift towards a slower phenotype (i.e. increased proportion of slow myofibres and decreased fast 2A and fast 2× myofibres) was observed at 29 months. Slow myofibre type groupings could also be clearly seen which is an indication of reoccurring myofibre denervation and re-innervation [4]. The shift to a slower phenotype is consistent with an earlier report for aged rat soleus muscle, with a loss of fast 2A myofibres and increased number of slow myofibres [32].

The conversion of myofibres from one histochemical type to another is possibly due to re-innervation by a different motoneuron type [59]. It is widely considered that fast type myofibre denervation is prevalent and that these become re-innervated by motoneuron axons that innervate slow type myofibres, which would then shift muscle phenotype to a slow oxidative profile [60]. However, our data suggest that the situation is more complex. Changes in myofibre type composition do not occur in a uniform pattern (e.g. shift from fast to slow) but rather are specific to individual muscles.

Our results in geriatric mice support the role of muscle denervation as a contributing factor to sarcopenia. We conclude that denervation of muscles is not due to the loss of motoneuron cell bodies, although it may result from degeneration or altered function of motoneuron axons. Morphological changes were observed in skeletal muscles of geriatric mice at the presynaptic nerve terminal, postsynaptic endplates and Schwann cells. The extent of age-related loss of muscle mass (sarcopenia) and alteration in myofibre types is specific to individual muscles: thus, results from one muscle type cannot be directly extrapolated to another within the same species. Establishing age-related baseline data in the neuromuscular compartment in mice provides a foundation for the use of murine models, with the wealth of genetically modified lines, to develop therapeutic interventions for sarcopenia.

## Methods

### Animals and tissue collection

All animal experiments were conducted in strict accordance with the guidelines of the National Health and Medical Research Council of Australia Code of Practice for the Care and Use of Animals for Scientific Purposes (2004) and the Animal Welfare Act of Western Australia (2002) and were approved by the Animal Ethics Committee at the University of Western Australia (Approval number 10/100/930).

Age-related neuromuscular changes were studied in 3 and 29 month old female C57BL/6J mice. Three month old mice were obtained from the Animal Resource Centre, Murdoch, Western Australia, and 29 month old mice were obtained from the Royal Brisbane hospital, Queensland, Australia. After transportation, mice were acclimatised for 1 week before tissues were taken. Mice were maintained in standard cages under pathogen-free conditions with free access to water and standard mouse chow.

Mice were anesthetised with a gaseous mixture of 1.5% Isoflurane (BioMac), N_2_O and O_2_, body weights recorded and animals sacrificed by severing the spine below the skull (C1–C2). Quadriceps, tibialis anterior (TA), extensor digitalis longus (EDL) and soleus muscles were excised from the hind limbs and weighed. TA muscles from both legs and EDL and soleus muscles from one leg were cut transversely, mounted on cork pieces with tragacanth gum (Sigma-Aldrich) and frozen in isopentane (BDH-AnalaR) cooled in liquid nitrogen for histological and immuno-histochemical analyses. EDL and soleus muscles from the other leg were fixed in 4% paraformaldehyde (PFA) in 0.1 Sorenson's phosphate buffer (0.084M Na_2_HPO_4_ and 0.016M NaH_2_PO_4_.2H_2_O) for 30 minutes at room temperature, then stored in Tris buffered saline (TBS) at 4°C until whole mount immuno-histochemistry was performed.

The first lumbar process of the spinal cord was identified by locating the first vertebra that lacked an articulation with a rib at its rostral margin [31]. The lumbar region (L1–L5) was excised, fixed in 4% PFA in phosphate buffer overnight, transferred to 30% sucrose, coated with Tissue-Trek O.C.T compound and frozen in isopentane cooled in liquid nitrogen for further cryo-sectioning.

### Motoneuron staining and analysis

The entire lumbar region of the spinal cord (L1–L5) was cryo-sectioned (Leica LM3050) at 20 µm (approximately 450 sections). Sections were collected onto Superfrost glass slides and stained with a solution of 0.05% Toluidine Blue and 0.005% Borax (pH11). Every 20^th^ section was analysed using Olympus BX50 microscope at ×20 magnification, starting from L1. Only neurons located in the ventro-lateral quarter of the spinal cord with a maximum diameter of ≥25 µm and a visible nucleolus were counted and measured and were presumed to be α-motoneurons (Fig. 1) [38], [61], [62], [63], [64]. We checked motoneurons in up to 20 sections from different animals at 3 and 29 months and found that all motoneurons examined had only one nucleolus. The maximum diameter of each α-motoneuron (i.e. ≥25 µm) drawn through the nucleolus was recorded and the total number of α-motoneurons counted [64], [65], [66], [67] (Fig. 1 A,B). Cell fragments, cells without visible nucleoli and motoneurons <25 µm in maximum diameter were excluded. Errors in identifying α-motoneurons may occur due to some overlap in sizes of α- and γ-motoneurons [15]. Although we may have overestimated the numbers of α-motoneurons, our identification and counting criteria were constant throughout our analyses. The α-motoneuron counts obtained do not represent the total number of α-motoneurons from L1–L5, but are the total number of motoneurons obtained from the 20 sections that we analysed over a distance of ∼450 µm.

### Whole mount immunohistochemistry to detect innervated and denervated NMJs and Schwann cells

To analyse the NMJs in EDL and soleus muscles, presynaptic nerve terminals were detected with synaptophysin antibody (Dako) and postsynaptic endplates with α-bungarotoxin (BTX: Invitrogen). Whole EDL and soleus muscles were blocked in 4% bovine serum albumin (Sigma) and 0.1% Triton X-100 (Roche) overnight at 4°C while rotating. Muscles were incubated with the primary rabbit-anti-synaptophysin antibody (1∶100 dilution) overnight at 4°C while rotating. Muscles were washed for 5 hours in TBS and incubated with the secondary antibody donkey-anti-rabbit IgG ALEXA 594 (Molecular Probes, 1∶250 dilution) and α-bungarotoxin ALEXA 488 (1∶1000 dilution) overnight at 4°C while rotating. Muscles were washed overnight and stored in glycerol until imaged. Prior to imaging, immunostained EDL and soleus muscles were flattened between two glass plates and imaged with a Leica TCS SP2 multiphoton confocal microscope. Nerve terminals stained with synaptophysin 594 (red) were detected with a 594 wavelength laser. Postsynaptic endplates on the myofibre stained with α-bungarotoxin 488 (green) were detected with the 488 wavelength laser. NMJs that had both red (synaptophysin) and green (BTX) staining were considered as innervated, while NMJs stained with only BTX were considered as denervated. Each selected field was imaged at ×20 magnification, at 2 µm per step, up to 100 µm and a Z-stacked image (∼50 images stacked together) was generated for each field of view. Approximately 10 fields were imaged per one EDL muscle which corresponds to 100–150 NMJs examined per animal. Schwann cells were identified with the rabbit anti-S100 (DakoCytomation, 1∶500 dilution) subsequently detected with the donkey anti-rabbit IgG ALEXA 594 secondary antibody (Invitrogen, 1∶500 dilution). The immunostaining procedure and the confocal imaging were the same as above.

### Muscle haematoxylin and eosin staining and imaging to quantitate total myofibre numbers and size

Transverse cryosections (8 µm) through the mid-region of the EDL and soleus muscles were stained with haematoxylin and eosin. Non-overlapping images were taken at ×10 magnification and tiled to reconstruct the cross-section of the muscle using a LEICA DMRBE microscope connected to a Nikon Digital Camera DXM1200F and Vexta stage movement software. Images were analysed with Image Pro Plus v4.5 (Microsoft) software.

### Identification of different myofibre types and morphometric analyses

Slow (MHCI) and fast 2A (MHCIIA) myofibres were identified with mouse-IgG1 antibodies against slow type myosin (Millipore, 1∶40 dilution) or fast MHCIIA type myosin (SC-71 supernatant, Developmental Studies Hybridoma Bank, 1∶5 dilution) that were conjugated to Zenon (Invitrogen) reagents for mouse IgG1 Alexa Fluor 594 (red) and Alexa Fluor 350 (blue) respectively. Fast 2B (MHCIIB) myofibres were identified with the mouse-IgM antibody against MHCIIB (BF-F3 supernatant, Developmental Studies Hybridoma Bank, 1∶5 dilution). The mouse anti-MHCIIB primary antibody was detected with the secondary goat anti-mouse IgM Alexa488 (Molecular Probes, 1∶250 dilution). Some myofibres were not stained with any of these antibodies and were presumed to be fast 2× (MHCIIX) [26]. Images were captured with a high resolution colour camera (Nikon digital camera DXM1200F) and imaging software (Nikon ACT-1 v 2.70), then analysed using ImagePro Plus v4.5 (Microsoft) software.

A single ×10 image per TA muscle was taken at the deeper region of the muscle closer to the tibial bone (inner portion) where slow myofibres are normally present in young (3 month old) mice, whereas the rest of the TA is predominantly fast [56]. In EDL and soleus muscles, a single random image was taken per muscle in the middle of the muscle cross-section. For images from each muscle, the number of slow type 1(red), fast 2A (blue), 2B (green) and 2× (unstained) myofibres were counted and expressed as a percent of total myofibre number. A total of approximately 150 to 300 myofibres were counted per muscle.

### Statistical Analysis

All data were analysed using Students t-test, 2 tailed, type 2 and expressed as mean ± standard error (s.e.m). Differences with P values<0.05 were considered significant.

## References

[1] Shavlakadze T, Grounds MD, (ed.) SISR, editor (2003). Therapeutic Interventions for Age-related Muscle Wasting - Importance of Innervation and Exercise for Preventing Sarcopenia.. Modulating Aging and Longevity Great Britian.

[2] Cruz-Jentoft AJ, Baeyens JP, Bauer JM, Boirie Y, Cederholm T (2010). Sarcopenia: European consensus on definition and diagnosis.. Age and Ageing.

[3] Baumgartner RN, Koehler KM, Gallagher D, Romero L, Heymsfield SB (1998). Epidemiology of sarcopenia among the elderly in New Mexico.. American Journal of Epidemiology.

[4] MacIntosh BR, Gardiner PF, McComasm AJ (2006). Skeletal muscle: form and function.

[5] Shavlakadze T, McGeachie J, Grounds MD (2010). Delayed but excellent myogenic stem cell response of regenerating geriatric skeletal muscles in mice.. Biogerontology.

[6] Lynch GS, Shavlakadze T, Grounds MD, R. SIS E (2005). Strategies to reduce age-related skeletal muscle wasting.. Ageing Interventions and Therapies.

[7] McMahon CD, Shavlakadze T, Grounds MD, Lynch GS (2011). Role of IGF-1 in Age-Related Loss of Skeletal Muscle Mass and Function..

[8] Altun M, Besche HC, Overkleeft HS, Piccirillo R, Edelmann MJ (2010). Muscle wasting in aged, sarcopenic rats is associated with enhanced activity of the ubiquitin proteasome pathway.. Journal of Biological Chemistry.

[9] Rennie MJ, Selby A, Atherton P, Smith K, Kumar V (2010). Facts, noise and wishful thinking: muscle protein turnover in aging and human disuse atrophy.. Scandinavian Journal of Medicine & Science in Sports.

[10] Rattan SIS (2010). Synthesis, modifications, and turnover of proteins during aging.. Experimental Gerontology.

[11] Thornell LE (2011). Sarcopenic obesity: satellite cells in the aging muscle.. Current Opinion in Clinical Nutrition & Metabolic Care.

[12] Luff AR (1998). Age-associated Changes in the innervation of muscle fibers and changes in the mechanical properties of motor units.. Annals of the New York Academy of Sciences.

[13] Flood DG, Coleman PD (1988). Neuron numbers and sizes in aging brain: Comparisons of human, monkey, and rodent data.. Neurobiology of Aging.

[14] Auld DS, Robitaille R (2003). Glial cells and ceurotransmission: an inclusive view of synaptic function.. Neuron.

[15] Tomlinson BE, Irving D (1977). The numbers of limb motor neurons in the human lumbosacral cord throughout life.. Journal of the Neurological Sciences.

[16] Knox CA, Kokmen E, Dyck PJ (1989). Morphometric Alteration of Rat Myelinated Fibers with Aging.. Journal of Neuropathology & Experimental Neurology.

[17] Valdez G, Tapia JC, Kang H, Clemenson GD, Gage FH (2010). Attenuation of age-related changes in mouse neuromuscular synapses by caloric restriction and exercise.. Proceedings of the National Academy of Sciences.

[18] Naguib M, Flood P, McArdle JJ, Brenner HR (2002). Advances in neurobiology of the neuromuscular junction: Implications for the anesthesiologist.. Anesthesiology.

[19] McLennan IS, Koishi K (2002). The transforming growth factor-betas: multifaceted regulators of the development and maintenance of skeletal muscles, motoneurons and Schwann cells.. The International Journal of Developmental Biology.

[20] Gambino DR, Malmgren LT, Gacek RR (1990). Age-related changes in the neuromuscular junctions in the human posterior cricoarytenoid muscles: A quantitative study.. The Laryngoscope.

[21] Arizono N, Koreto O, Iwai Y, Hidaka T, Takeoka O (1984). Morphometric analysis of human neuromuscular junction in different ages.. Pathology International.

[22] Dedkov EI, Kostrominova TY, Borisov AB, Carlson BM (2003). MyoD and myogenin protein expression in skeletal muscles of senile rats.. Cell and Tissue Research.

[23] Courtney J, Steinbach JH (1981). Age changes in neuromuscular junction morphology and acetylcholine receptor distribution on rat skeletal muscle fibres.. The Journal of Physiology.

[24] Jang YC, Lustgarten MS, Liu Y, Muller FL, Bhattacharya A (2010). Increased superoxide in vivo accelerates age-associated muscle atrophy through mitochondrial dysfunction and neuromuscular junction degeneration.. The Journal of the Federation of American Societies for Experimental Biology.

[25] The Jackson Laboratory (2011).

[26] Schiaffino S, Gorza L, Sartore S, Saggin L, Ausoni S (1989). Three myosin heavy chain isoforms in type 2 skeletal muscle fibres.. J Muscle Res Cell Motil.

[27] Frontera WR, Hughes VA, Fielding RA, Fiatarone MA, Evans WJ (2000). Aging of skeletal muscle: a 12-yr longitudinal study.. Journal of Applied Physiology.

[28] Aniansson A, Hedberg M, Henning G-B, Grimby G (1986). Muscle morphology, enzymatic activity, and muscle strength in elderly men: A follow-up study.. Muscle & Nerve.

[29] Edström E, Altun M, Bergman E, Johnson H, Kullberg S (2007). Factors contributing to neuromuscular impairment and sarcopenia during aging.. Physiology & Behavior.

[30] Aagaard P, Suetta C, Caserotti P, Magnusson SP, Kjaer M (2010). Role of the nervous system in sarcopenia and muscle atrophy with aging: strength training as a countermeasure.. Scandinavian Journal of Medicine & Science in Sports.

[31] Rigaud M, Gemes G, Barabas M-E, Chernoff DI, Abram SE (2008). Species and strain differences in rodent sciatic nerve anatomy: Implications for studies of neuropathic pain.. Pain.

[32] Caccia MR, Harris JB, Johnson MA (1979). Morphology and physiology of skeletal muscle in aging rodents.. Muscle & Nerve.

[33] Stanmore A, Bradbury S, Weddell AG (1978). A quantitative study of peripheral nerve fibres in the mouse following the administration of drugs. 1. Age changes in untreated CBA mice from 3 to 21 months of age.. Journal of Anatomy.

[34] Gutmann E, Hanzlkova V (1966). Motor unit in old age.. Nature.

[35] Kwon BK, Liu J, Messerer C, Kobayashi NR, McGraw J (2002). Survival and regeneration of rubrospinal neurons 1 year after spinal cord injury.. Proceedings of the National Academy of Sciences of the United States of America.

[36] Fujisawa K (1976). Some observations on the skeletal musculature of aged rats–III. Abnormalities of terminal axons found in motor end-plates.. Experimental Gerontology.

[37] Ulfhake B, Bergman E, Edstrom E, Fundin B, Johnson H (2000). Regulation of neurotrophin signaling in aging sensory and motoneurons.. Molecular Neurobiology.

[38] Jacob JM (1998). Lumbar motor neuron size and number is affected by age in male F344 rats.. Mechanisms of Ageing and Development.

[39] Morrison JH, Hof PR (1997). Life and Death of Neurons in the Aging Brain.. Science.

[40] O'Donnell KA, Rapp PR, Hof PR (1999). Preservation of Prefrontal Cortical Volume in Behaviorally Characterized Aged Macaque Monkeys.. Experimental Neurology.

[41] Wang Z-M, Zheng Z, Messi ML, Delbono O (2005). Extension and magnitude of denervation in skeletal muscle from ageing mice.. The Journal of Physiology.

[42] Deschenes MR, Roby MA, Eason MK, Harris MB (2010). Remodeling of the neuromuscular junction precedes sarcopenia related alterations in myofibers.. Experimental Gerontology.

[43] Jang YC, Van Remmen H (2011). Age-associated alterations of the neuromuscular junction.. Experimental Gerontology.

[44] Kawabuchi M, Zhou CJ, Wang S, Nakamura K, Liu WT (2001). The spatiotemporal relationship among schwann cells, axons and postsynaptic acetylcholine receptor regions during muscle reinnervation in aged rats.. The anatomical record.

[45] Ludatscher RM, Silbermann M, Gershon D, Reznick A (1985). Evidence of schwann cell degeneration in the aging mouse motor end-plate region.. Experimental Gerontology.

[46] Wokke JHJ, Jennekens FGI, van den Oord CJM, Veldman H, Smit LME (1990). Morphological changes in the human end plate with age.. Journal of the Neurological Sciences.

[47] Bunge RP (1994). The role of the Schwann cell in trophic support and regeneration.. Journal of Neurology.

[48] Kawabuchi M, Chongjian Z, Islam ATMS, Hirata K, Nada O (1998). The effect of aging on the morphological nerve changes during muscle reinnervation after nerve crush.. Restorative Neurology and Neuroscience.

[49] Arthur PG, Grounds MD, Shavlakadze T (2008). Oxidative stress as a therapeutic target during muscle wasting: considering the complex interactions.. Curr Opin Clin Nutr Metab Care.

[50] Gomez-Cabrera M-C, Domenech E, Viña J (2008). Moderate exercise is an antioxidant: Upregulation of antioxidant genes by training.. Free Radical Biology and Medicine.

[51] Rowe RWD (1969). The effect of senility on skeletal muscles in the mouse.. Experimental Gerontology.

[52] Lexell J, Taylor CC, Sjöström M (1988). What is the cause of the ageing atrophy?: Total number, size and proportion of different fiber types studied in whole vastus lateralis muscle from 15- to 83-year-old men.. Journal of the Neurological Sciences.

[53] Fahim MA, Robbins N (1982). Ultrastructural studies of young and old mouse neuromuscular junctions.. Journal of Neurocytology.

[54] Sheard PW, Anderson RD (2011). Age-related loss of muscle fibres is highly variable amongst mouse skeletal muscles.. Biogerontology.

[55] Shavlakadze T, White JD, Davies M, Hoh JFY, Grounds MD (2005). Insulin-like growth factor I slows the rate of denervation induced skeletal muscle atrophy.. Neuromuscular Disorders.

[56] Parry DJ, Wilkinson RS (1990). The effect of reinnervation on the distribution of muscle fibre types in the tibialis anterior muscle of the mouse.. Can J Physiol Pharmacol.

[57] Augusto V, Padovani CR, Campos GER (2004). Skeletal muscle fiber types in C57Bl6J mice.. Brazil Journal of Morphology Science.

[58] Wigston DJ, English AW (1992). Fiber-type proportions in mammalian soleus muscle during postnatal development.. Journal of Neurobiology.

[59] Klitgaard H, Zhou M, Schuaffino S, Betto R, Salviati G (1990). Ageing alters the myosin heavy chain composition of single fibres from human skeletal muscle.. Acta Physiologica Scandinavica.

[60] Grimby G, Danneskiold-Samsoe B, Hvid K, Saltin B (1982). Morphology and enzymatic capacity in arm and leg muscles in 78–81 year old men and women.. Acta Physiologica Scandinavica.

[61] McHanwell S, Biscoe TJ (1981). The localization of motoneurons supplying the hindlim muscles of the mouse.. Philosophical Transactions of the Royal Society of London Series B, Biological Sciences.

[62] Gadamski R (2006). Morphological changes and selective loss of motoneurons in the lumbar part of the spinal cord in a rat model of familial amyotrophic lateral sclerosis (fALS).. Folia Neuropathologica.

[63] Stephens B, Guiloff RJ, Navarrete R, Newman P, Nikhar N (2006). Widespread loss of neuronal populations in the spinal ventral horn in sporadic motor neuron disease. A morphometric study.. Journal of the Neurological Sciences.

[64] Vercelli A, Mereuta OM, Garbossa D, Muraca G, Mareschi K (2008). Human mesenchymal stem cell transplantation extends survival, improves motor performance and decreases neuroinflammation in mouse model of amyotrophic lateral sclerosis.. Neurobiology of Disease.

[65] West MJ, Slomianka L, Gundersen HJ (1991). Unbiased stereological estimation of the total number of neurons in thesubdivisions of the rat hippocampus using the optical fractionator.. The Anatomical Record.

[66] Ciavarro GL, Calvaresi N, Botturi A, Bendotti C, Andreoni G (2003). The densitometric physical fractionator for counting neuronal populations: application to a mouse model of familial amyotrophic lateral sclerosis.. Journal of Neuroscience Methods.

[67] Coggeshall RE, Lekan HA (1996). Methods for determining numbers of cells and synapses: A case for more uniform standards of review.. The Journal of Comparative Neurology.

